# Homeostatic Left Heart integration and disintegration links atrio-ventricular covariation’s dyshomeostasis in Hypertrophic Cardiomyopathy

**DOI:** 10.1038/s41598-017-06189-w

**Published:** 2017-07-24

**Authors:** Paolo Piras, Concetta Torromeo, Antonietta Evangelista, Stefano Gabriele, Giuseppe Esposito, Paola Nardinocchi, Luciano Teresi, Andrea Madeo, Michele Schiariti, Valerio Varano, Paolo Emilio Puddu

**Affiliations:** 1 Dipartimento di Scienze Cardiovascolari, Respiratorie, Nefrologiche, Anestesiologiche e Geriatriche, Sapienza – Università di Roma, Rome, Italy; 2 Dipartimento di Ingegneria Strutturale e Geotecnica, Sapienza – Università di Roma, Rome, Italy; 3 Ospedale San Giovanni Calibita Fatebenefratelli – Isola Tiberina, Rome, Italy; 4 Dipartimento di Architettura, LaMS – Modeling & Simulation Lab, Università Roma Tre, Rome, Italy; 5 Dipartimento di Matematica e Fisica, LaMS – Modeling & Simulation Lab, Università Roma Tre, Rome, Italy

## Abstract

Left ventricle and left atrium are and have been practically always analyzed separately in common clinically and non-clinically oriented cardiovascular investigations. Both classic and speckle tracking echocardiographic data contributed to the knowledge about deformational impairments occurring in systo-diastolic differences. Recently new trajectory based approaches allowed a greater awareness about the entire left ventricle or left atrium revolution and on their deficiencies that take place in presence of hypertrophic cardiomyopathy. However, surprisingly, the concomitant function of the two left heart chambers has not been analyzed for their geometrical/mechanical relationship. For the first time we study here, by acquiring left ventricle and left atrial geometries on the same heartbeat, the trajectory attributes of the entire left heart treated as a whole shape and the shape covariation of its two subunits. We contrasted healthy subjects with patients affected by hypertrophic cardiomyopathy. We found impaired left heart trajectory mainly in terms of orientation and size. More importantly, we found profound differences in the direction of morphological covariation of left ventricle and left atrium. These findings open to new perspectives in pathophysiological evaluation of different diseases by allowing the appreciation of concomitant functioning of both left heart whole geometry and of its two chambers.

## Introduction

In recent years, the use of three-dimensional analyses in cardiological diagnosis and pathophysiological investigations became very common in scientific literature^1–5^. In particular, easy accessibility, non-invasive approach and fast acquisition of 2D and 3D Speckle Tracking Echocardiography (2DSTE, 3DSTE) made these tools among the most common instruments in cardiology^6–10^. Alternatively, Cardiac Magnetic Resonance (CMR) can be used to acquire precise heart geometry during its functioning^11–13^. However, CMR is much more invasive than 2DSTE or 3DSTE and does not enable the concomitant acquisition of electrical heart activity. A large amount of scientific literature (whose complete review if far beyond the scope of the present paper) investigated a variety of pathologies and highlighted geometrical and mechanical properties of different diseases by means of 3DSTE whose output variables comprehend different mean strain measures^14–20^. Recently, a new approach was proposed, based on the analysis of the landmarks’ cloud upon which 3DSTE strains are computed, so that heart deformation during its revolution in physiological and patho-physiological states can be easily investigated^21–25^. Thus, translational contributions of differential geometry and clinical cardiology led to the application of Parallel Transport (PT) to atrial or ventricular geometries aimed at studying pure deformational patterns irrespective of the actual starting inter-individual heart shape differences^3, 8, 14, 16^. Exploration of not only *deformation per se* but also of the shape of heart trajectory itself was therefore possible to provide a precise characterization of the *motion trajectory per se*. This is conceivable trough the introduction of temporal homology’ notion. Heart shapes are then evaluated (via spline-interpolation) at specific electro-mechanical homologous times such as R peak of the electrocardiogram or mitral valve opening (among others). Using common ordination methods, such as Principal Components Analysis (PCA), the “landmarks” of a trajectory shape are represented by the coordinates of dominant PC scores (PCs) i.e. the main deformation modes.

One of the most salient features of the abovementioned studies was that they focused on left ventricle (LV) or on left atrium (LA) only. To the best of our knowledge, there are no simultaneous investigations on the two chambers together which explore the Left Heart (LH) in its entirety. On the other hand, LV and LA indissolubly *covary* one with each other during cardiac revolution, hence forming LH dynamical change in time. Moreover, the entire LH can be viewed in two different ways: a structure composed of two, highly integrated, sub-units (LV and LA) and a sole one structure having its own shape and its own motion trajectory that can be studied with methods briefly explained above.

Here we focus on hypertrophic cardiomyopathy (HCM) and we show how LH’ investigation introduce new directions in studying heart diseases. HCM is the most common genetically-based heart disease with dominant autosomal transmission and complete or incomplete penetrance although it presents a highly variable phenotypic, clinical and prognostic heterogeneity^26^ and about 1400 mutations carried by more than 13 genes were identified^27, 28^. The attributes of HCM trajectory (its orientation, shape and size) appeared to be very effective in recognizing HCM for both LV^16^ and LA^8^. Here, we first hypothesize that the entire LH presents impaired motion attributes in HCM. Then, we hypothesize that the integration of the two LV and LA subunits, i.e. the way they covary in time, is also impaired. In particular, we investigate two attributes of morphological integration, strength and direction, trough recently proposed techniques exploiting Partial Least Squares analysis (PLS) capabilities^29^. Eventually, we also explore the covariation patterns between the shapes of LV and LA trajectories.

## Results

### Reproducibility

Intra-observer variability analysis shows that the reproducibility of our results is always very high (smaller than 2%). Traditional global 3DSTE parameters of the left ventricle show a very good coefficient of variation: Volume: 1.56%; Radial Strain global: 0.84%; Circumferential Strain global: 0.79%; Longitudinal Strain global: 0.81%; Rotation global: 1.18%; Twist global: 0.96%; Torsion Regional global: 0.84%; Torsion Basal global: 0.96%; Strain 3D global: 0.79%; Radial Displacement global 0.64%; Longitudinal Displacement global 1%. The same emerges from the distances between cycles shape replicas of the same subject parameterized on pi/2: mean: 0.00991; sd: 0.01605. Inter-observer variability returned also a very good performance being coefficient of variation of traditional global 3DSTE parameters of the left ventricle always smaller than 5%.

### Left Heart trajectory

**Figure 1:**
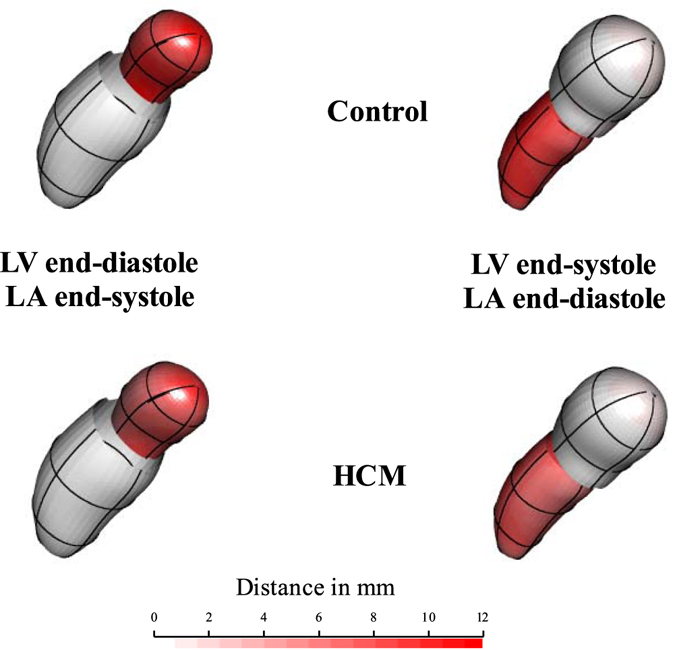
Global deformational patterns in Control and HCM. The colormap ranges from white (min) to red (max) and refers to |x*M* − x|, with x*M* the position of a point in the LV or LA diastole and x the position of the corresponding deformed state. This was done here just for sake of visualization as the Common Template from which deformation were computed was chosen in correspondence of the Grand Mean of all time frames of all cardiac cycles instead that in correspondence of a particular deformed/undeformed state. See text for further explanation. Supplementary Figure S1 shows the corresponding animation in full color using blue-cyan-yellow-red color palette.

Figure 1 shows the end-systolic and end-diastolic states according to PT analysis performed on the endocardial surfaces of LV and LA (see Material and Methods). Supplementary Figure S1 shows the corresponding animation in full color using blue-cyan-yellow-red color palette. For sake of visualization the colormap represents the Euclidean distance from the end diastolic state for LV and the end-systolic state for LA. It is evident that Control deforms more than HCM in both ventricular and atrial contractions.

**Figure 2:**
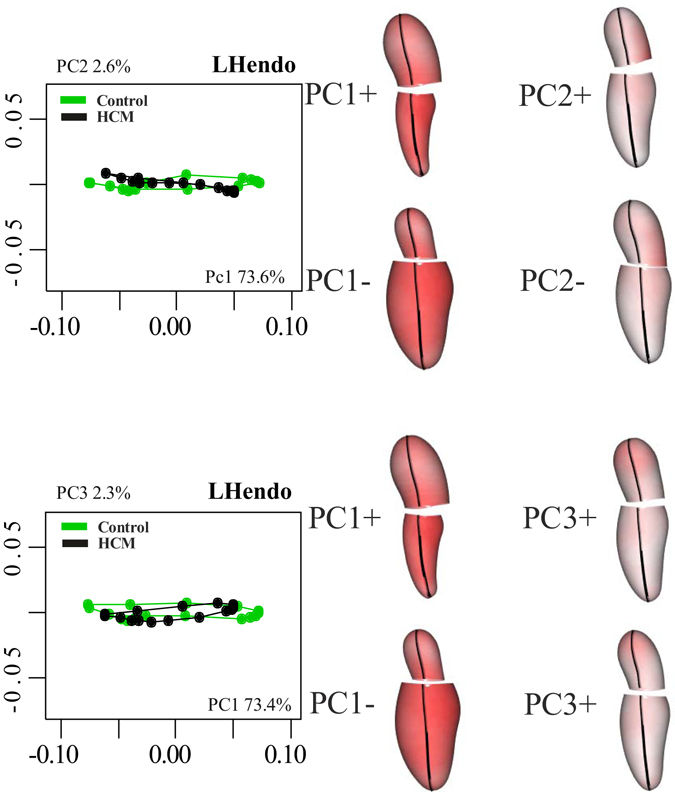
PCA on shapes after Parallel Transport. Centering deformations on GM (differently from what visualized in Fig. 1) allows to consider both systole and diastole as deformed states. Deformations relative to the GM are depicted. The colormap ranges from white (min) to red (max) and refers to |x*M* − x|, with x*M* the position of a point in the GM and x its position at the specified PC axis extreme (positive or negative). The first three PCs are shown. Explained variances (in percentage) are also indicated.

Figure 2 shows per-group mean LH trajectories in the PCA space. Supplementary Figure S2 shows the trajectories in the space of the first three PCs with fitting planes for each category in the shape space. Trajectories look different in extension and orientation and this is evidenced by data distribution at each homologous time for each of the first three PCs.

**Figure 3:**
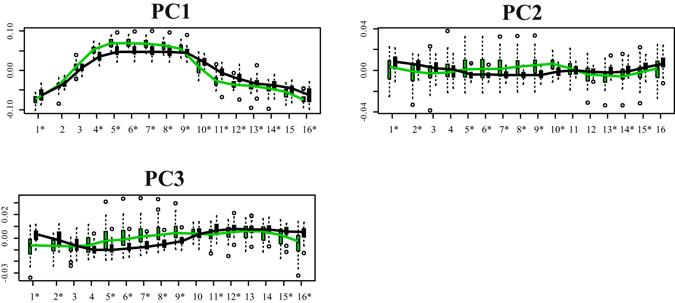
Distribution of the first three PC scores among Control (green) and HCM (black). Boxplots relative to Control and HCM are displayed at all homologous times. Asterisks indicate significance under ANOVA. As it can been seen, even the diastolic state significantly differs between the two categories.

Figure 3, in fact, shows the boxplot distribution of dominant PCs for Control and HCM. The two categories significantly differ at virtually all homologous times (asterisks in the ordinal time positions designate significance) indicating strong differences in location. PC1 explains a large fraction of total variance (about 73%). As our analyses are performed in the shape space (thus filtering out size), this means that, besides size, the large part of deformation is concentrated along a single axis.

**Figure 4:**
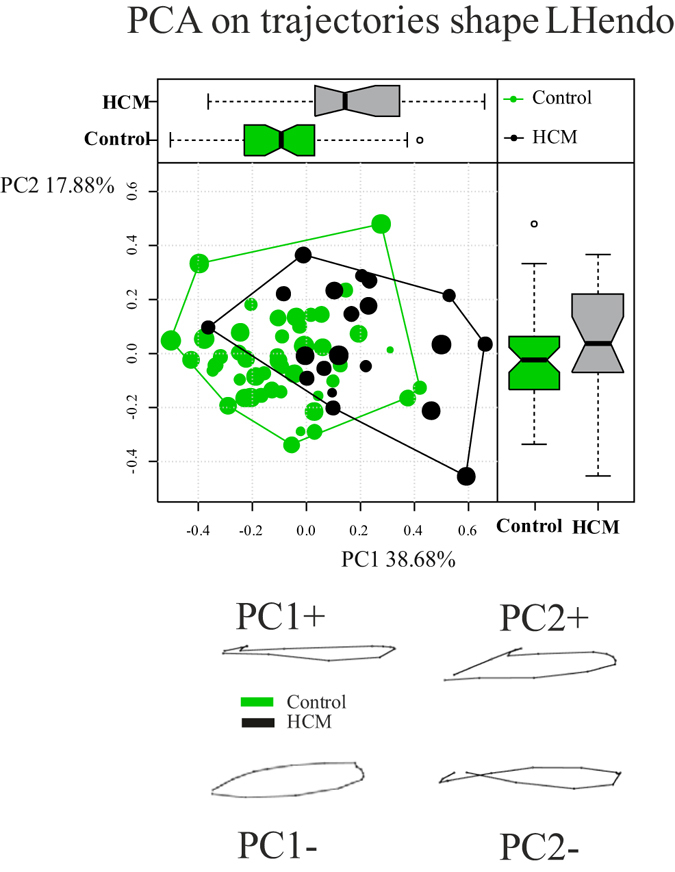
Shape analysis on trajectories shapes. Both PC1/PC2 scatterplot and boxplot distributions for each PC are shown. Trajectories shapes refer to PC extreme values.

Figure 4 shows results of GPA + PCA performed on trajectories shapes identified by the first three PCs of LS analysis. Among dominant PCs only the first one significantly separates Control from HCM (R-sq: 0.24; *p*-value: 0.00002). At positive PC1 values, were HCM places, the trajectory is flat while at negative ones, where Control stays, the trajectory shape is more rounded.

Results relative to size and orientation of LH trajectory highlights that Control presents a significant larger size in comparison to HCM (mean Control: 0.224; mean HCM: 0.17; Anova R-sq: 0.23; *p*-value: 3.9e-5) as well as a significant different orientation of both angles (PC1/PC2 angle: mean Control: 0.92; mean HCM: −5.74; Anova R-sq: 0.11; *p*-value: 0.003; PC1/PC3 angle: mean Control: 87.7; mean HCM: −95.7; Anova R-sq: 0.22; *p*-value: 4.9e-5).

### Integration and disintegration

**Figure 5:**
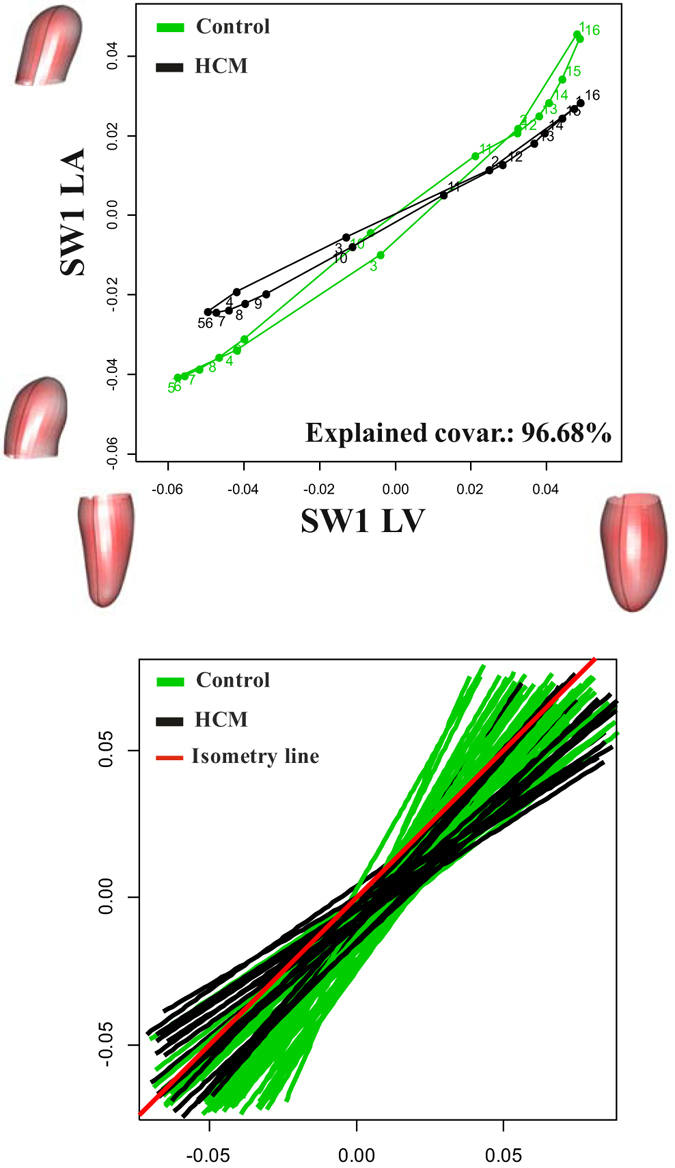
Shape covariation between LV and LA. Mean covariation trajectories (top) and MA regression fitting lines (bottom) are shown. Shapes correspond to extreme values (positive or negative) of Singular Warps axes.

The mean slopes of disintegration analysis do not differ among groups (Control: −2.23; HCM: −2.17; *p*-value: 0.151). These values indicate that LH is, as expected, a highly integrated structure when taken as a whole shape and that the two categories show the same degree of integration. A coherent result is found when using PLS on LV and LA blocks. Figure 5 shows mean covariation trajectories and associated shapes as well as the stacks of MA regression lines for each individual performed on the model SW1_LA_~SW1_LV_.

Results relative to the strength and direction of covariation show that strenght’s z-scores do not significantly differ in the two groups (mean Control: 5.09; mean HCM: 5.03; Anova R-sq: 0.007; *p*-value: 0.49), while the directions are very different as illustrated by mean covariation trajectories and MA slope stacks (mean Control: 1.24; mean HCM: 0.88; Anova R-sq: 0.23; *p*-value: 4.36e-5; see Fig. 5). It is important to note that Control shows a mean slope larger than 1, i.e. isometry, (1.25), while HCM have a smaller value (0.89). This indicates that in Control, LA shape change per unit of LV shape change is significantly larger than that of LV whereas it is the opposite for HCM.

### LV-LA trajectories covariation

**Figure 6:**
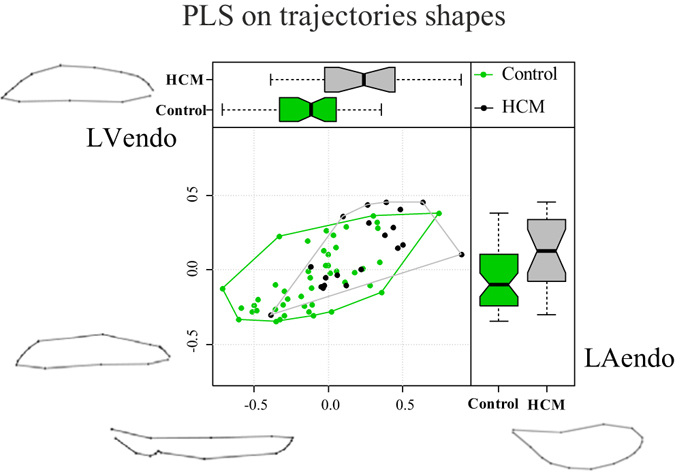
Covariation between LV and LA trajectories shapes.

When PLS analysis was applied to the shapes of trajectories themselves we found no significant differences in both strength and direction of covariation (PLS effect size: 1.33; *p*-value: 0.09; Control slope: 1.44; HCM slope: 1.26; *p*-value: 0.61). Figure 6 shows the first pair of SW axes that explains 66.6% of total covariance.

### Clinical classification

Table 1 summarizes the results relative to classification power of the various LH deformational parameters computed in this study as well as those relative to the most common 3DSTE parameters. Among these, the LA volume index is a measure of pure size and it is not directly comparable to our indicators that are computed in the Shape Space. Moreover, the LV mass strongly depends on the interventricular septum thickness that is the parameter upon which HCM pathology is recognized and it is therefore affected by severe circular reasoning.

**Table 1:** Classification performance of new and traditional indicators according to randomized SVM.

Independent variables	AUC	Total accurracy	Specificity	Sensitivity
***Newly proposed parameters***				
LS PC1 at all homologous times	0.88	0.86	0.93	0.60
LS PC2 at all homologous times	0.84	0.77	0.82	0.56
LS PC2 at all homologous times	0.80	0.77	0.84	0.52
LS PC1/PC2/PC3 at all homologous times	0.89	0.91	0.96	0.72
LH trajectory size	0.79	0.81	0.93	0.39
LH trajectory PC1/PC2 angle	0.64	0.72	0.89	0.10
LH trajectory PC1/PC3 angle	0.81	0.81	0.90	0.48
LH trajectory shape first 10 PCs	0.77	0.80	0.88	0.50
LV-LA covariation direction	0.70	0.81	0.93	0.39
***Traditional 3DSTE parameters***				
*3D Left ventricular mass	0.96	0.95	0.97	0.85
*Indexed 3D Left ventricular mass	0.93	0.94	0.97	0.84
**3D Left atrium Volume indexed	0.86	0.92	0.97	0.73
Left atrium radial global strain	0.62	0.74	0.93	0.02
Left atrium circumferential global strain	0.74	0.84	0.95	0.45
Left atrium longitudinal global strain	0.69	0.72	0.83	0.32
Left ventricle radial global strain	0.64	0.74	0.92	0.07
Left ventricle circumferential global strain	0.69	0.78	0.93	0.22
Left ventricle longitudinal global strain	0.78	0.80	0.91	0.39

Asterisk indicates that circular reasoning affects corresponding results. Double asterisk indicates a size-associated parameter.

LS PC1 shows a very good AUC and total accuracy as well as the second value of sensitivity. The best values are found for the first three LS PCs combined together. Among trajectory attributes the PC1/PC3 angle shows the best performance. Trajectory size and shape perform satisfactorily as well, except for sensitivity. Our parameters perform very well when compared to the traditional 3DSTE measures when circular reasoning affected and scale-connected parameters are ignored. In general, the worst values are those of sensitivity due to the unbalanced design

## Discussion

To our best knowledge we presented the first combined analysis of functional covariation of LV and LA as well as the investigation of the entire LH treated as a whole shape. The analysis applied to the comparison of Control and HCM allowed to augment our knowledge about HCM pathophysiology which had been investigated solely at the one-chamber-level of LV^16^ or LA^8^. LS analysis showed, according to previous investigations^8, 16^, that the contraction measured in terms of Euclidean distance is smaller in HCM. This is evident in Fig. 1, looking at the colormap, and in the trajectory space illustrated in Fig. 2 where HCM occupies a smaller region of the morphospace along PC1, corresponding to the alternating systo-diastolic states of LV and LA. The fact that our analyses are performed in SS and that PC1 explains a large part of total variance (about 74%) indicates that, irrespective of size, the shape change follows a single direction dictated by LV-LA alternate contraction. Along PC1 axis the majority of affine (non-homothetic) and non-affine deformations occur. PC2 seems to indicate a shear-like deformation of LA shape associated to a change in the slope of mitral annulus’ border. Similar deformations occur, to some extent, along PC3. At virtually all homologous times, the first three PCs clearly discriminate Control from HCM mainly in correspondence of LV end-systole. However, PC3 shows a neat separation also at LV end-diastole (that coincides with LA end-systole) as shown in Fig. 3. The analysis of trajectories attributes indicates that only PC1 of the shape analysis significantly separates Control from HCM. Control shows a more rounded trajectory in comparison to HCM. The smaller size of HCM is consistent with the observed minor deformation illustrated in Fig. 1; the latter indicates that LH systole and diastole occur in different regions of LS morphospace for Control and HCM. While these evidences had been already noted for LV^16^ and LA^8^ alone, LH’ angles impairment suggests that the covariation of the two chambers could be impaired too. On the one hand, the strength of covariation is not different between Control and HCM as shown by disintegration analysis and PLS. On the other hand, the *direction* of covariation is significantly different among the two categories as evidenced by MA analysis and slope directions illustrated in Fig. 5. This suggests that LV and LA concomitant beat points toward an impaired trend in HCM. MA highlighted that, under the individual-specific model SW1_LA_~SW1_LV_, the rate of LA shape change per unit of LV shape change is higher in Control with a mean slope larger than 1 (isometry). The opposite holds for HCM with a mean slope smaller than 1. This result deserves special attention. As the analyses were performed in SS, any aspects of pure size are filtered out differently from what done previously^3, 8, 14, 16^. The higher rate of LA shape change per LV shape change in Control leads to the conclusion that the non-homothetic affine (or *deviatoric* in continuum mechanics vocabulary) and non-affine components of shape change are larger in LA than in LV that is more dominated by size. The reverse happens in HCM. It is well known^8, 30^ that in HCM the LA is hyper-dilated and that its ejection fraction is reduced. On the opposite, the absolute booster pump function is significantly augmented. It means that the pure volumetric component becomes more important in HCM’s LA, while the deviatoric and non-affine components turn out to be less significant.

The deformational indicators we presented in this study show a very good classification power (Table 1). The best performance is carried by the combination of the first three LS PC scores. PC1/PC3 angle shows also a very good performance. Sensitivity, however, does not reach very high values such as total accuracy or specificity. This could be explained, at least in part, by the unbalanced design, the small HCM sample size and the variance of pathology severity. Challenging our approach by comparing the above mentioned classification results with those coming from traditional 3DSTE parameters and, above all, LV mass and LA volume indexes, corroborates the goodness of our new methodology in searching for LH impairment in HCM. In addition, we stress that, as we treat LH as a whole shape, there are no directly comparable measures in the literature as only LV or LA deformations are usually studied without considering their functional covariance as in here.

In order to derive potentially useful and/or applicable conclusions for clinics it is important to underline how *shape* and *size-and-shape* might evolve in transition from healthy to pathological condition and specifically how LA-LV mechanical covariation behaves. Indeed, healthy LV is dominated by size whereas in presence of shape changes there might be a rapid evolution towards pathology^31^. LA might be instead more compliant in terms of both size and shape and we had shown^8^ that in HCM the pure size component is augmented as expected, provided its absolute booster pump function is concomitantly increased^30^.

Architectural, cellular and molecular structures are intimately connected to heart form in accordance to Laplace’s law: LV wall size and elastance compensate for LV internal radius and related end-diastolic volume (EDV), thus minimizing LV end-diastolic pressure. So it is tempting to speculate that the above mentioned structures are normal determinants of cardiac performance whose function is regulated by physiological and biochemical mechanisms. When these mechanisms fail, LV form and function are no more correlated to each other as in the normal state. As a consequence, LA follows LV impairment in HCM. This has two different outcomes: the strength of the two chamber’s covariation is maintained and the direction of this covariation is impaired. The first evidence must be interpreted in terms of *functional homeostasis* of heart organ that, while functioning in a living subject, cannot deviate from a given covariation constraint even in diseased conditions whose departure could be incompatible with life. The second one indicates clearly that this functional homeostasis cannot occur without a *cost*.

Following Kamalov *et al*.^32^, it was Claude Bernard to hypothesize the “homeostasis” concept in 1865: he postulated that the body possesses an intrinsic capability to preserve its *milieu interieur*, i.e. its internal stability in front of a perturbation^33^. Successively, Cannon^34^ presented the term “homeostasis” to meet original Bernard’s concept of internal stability. Quoting^32^ “Homeostasis reflects the body’s natural power of adaptation, i.e. a continuous cooperation and integration between the internal environment with external surroundings”. Very often the concept of homeostasis is referred to temperature, or hormone and ion concentration. We tentatively try to use it by referring to the structural and geometric compensation that LA experiences in presence of HCM augmented LV filling pressure. In HCM, in fact, LA pump function is increased to help ventricular filling. As mentioned above, while the maintenance of the strength of covariation could be interpreted in terms of functional homeostasis, the fact that we found a shift in its direction could be interpreted in terms of functional “dyshomeostasis” following Kamalov *et al*.’s vocabulary^32^. Dyshomeostasis can be defined as an inappropriate, deficient or excessive counter-regulation in response to the perturbation. The differences in the direction of covariation we found in this study can fall into these categories. The changing in slope could lead, in extreme conditions, to a potentially lethal state thus making this dyshomeostasis fatal. For example, other types of pathologies, such as aortic regurgitation or congestive heart failure, might present different, possibly more dyshomeostatic, LV-LA covariations. Another example of functional homeostasis followed by dyshomeostasis can be found in^35^ where the authors, studying HCM, found an increased duration of LV twist consequent to the inverse relationship of longitudinal strain rate and twist rate. This is viewed as a compensation ( = functional homeostasis) to preserve ejection fraction ( = systolic function). However, the increased duration of systolic twist induces an untwisting delay, with elevated LV early diastolic pressures, reduced transmitral pressure gradient and impaired LV early diastolic filling ( = dyshomeostasis). More in general, referring to other diseases different from HCM, our results suggest that the pure size and shape change evaluated not only in terms of classical 3DSTE parameters (i.e. Twist, Strain, Rotation, etc.) could provide important descriptors for interpreting not only frank pathology but also incumbent disease. While the volumetric indicator, either atrial or ventricular, has been always used to describe a great number of pathologies^36, 37^ in terms of ejection fraction, the new era of echocardiography, represented by 2DSTE and 3DSTE, introduced the possibility to study a large number of traditional-echocardiographic and new-deformational parameters. However, this frequently becomes a sort of boomerang as the huge plethora of parameters is often difficult to present, interpret and, above all, to export in common clinical practice. Coupling the main deformational parameters with the synthetic ones we proposed here and using them for describing the whole LH functioning and covariation could help in interpreting pathology and its mechanical and geometry-driven functional consequences.

## Material and Methods

### Data acquisition

Following^8, 14, 16^ we acquired data using PST–25SX Artida device, Toshiba Medical Systems Corp., Tokyo, Japan. We enrolled a total of 74 Control subjects found healthy after an accurate cardiological visit and 32 HCM patients. We then excluded all acquisitions with poor image quality and with ultrasonic windows not including completely both the left atrium and the left ventricle. After this selection the Control group was constituted by 46 subjects and 20 HCM patients (62.2% and 62.5% of total acquisitions, respectively). Inclusion criteria for HCM patients were determined by a left ventricular septum having a thickness larger than 13 mm. Table 2 reports details and descriptive statistics for the population under study.

**Table 2:** Descriptive statistics ± standard deviation for the populations under study.

**Descriptive parameters**	Control = 46	HCM = 20
Age (years)	39.2 ± 8.33	48.6 ± 12.61
Ejection Fraction (%)	59.1 ± 0.05	54.8 ± 0.08
Inter-Ventricular Septum (mm)	8.47 ± 1.43	18.2 ± 4.03
Males/Females	30/16	13/7
Beat rate (beat/s)	77 ± 13.16	76 ± 13.00

LH, constituted by LV and LA geometries, was reconstructed starting from 6 homologous landmarks for LV and 6 for LA. These were manually identified by the operator for each subject. The same operator (AE) was involved in LH reconstruction. The final geometry of any subject is a time-sequence of 16 shapes (see the homologous times interpolation method below), each one constituted by 2594 landmarks (presumed to be homologous) for endocardial surface, including LV and LA. LV and LA are individually composed by 1297 landmarks positioned along 36 horizontal circles, each composed of 36 landmarks, plus the apex. It is critical to specify here that LV and LA were digitized on the *same* 3DSTE clip. This ensures that the two LH chambers are acquired during the same heart beat and according to their reciprocal anatomical position and concomitant function. It was possible to obtain the landmark cloud (upon which the standard rotational, torsional and strain parameters are computed and outputted by each Artida device) by an unlocked version of the software equipping our PST–25SX Artida device, thanks to a special opportunity provided in the context of an official research and development agreement between the Dipartimento di Scienze Cardiovascolari, Respiratorie, Nefrologiche Anestesiologiche e Geriatriche, “Sapienza” Università di Roma and Toshiba Medical System Europe, Zoetermeer, The Netherland.

### Reproducibility

As the same operator (AE) was involved in geometry reconstructions, we performed an intra-observer reproducibility analysis. The left ventricles of 7 randomly chosen Controls and 3 (randomly chosen) HCM patients were reconstructed twice at a temporal distance >1 year (long-term). As first, we calculated the coefficient of variation of traditional 3DSTE global strains and volumes of left ventricles that possess larger systo-diastolic differences in comparison to left atrium using not only the systolic values but also those corresponding to all frames acquired by the device during one cycle. Then, as we propose here some novel indicators built using the Geometric Morphometrics paradigm (see below), we applied to each replica’s couple of acquisitions the deformation analysis presented below. We then evaluated the shape distance (via Procrustes distance, see below) between cycle’ shapes (using the first three PC scores as explained below) of each replica of the same subject parameterized on the maximum value it can assume on the tangent space that is pi/2. Coefficient of variation in percentage (i.e. standard deviation divided by the mean*100) applied to absolute difference between the two replicas of each subject for the global 3DSTE parameters was used as measure of goodness for reproducibility of classical 3DSTE variables. Tentatively one of us (GE) digitized the same subjects used for intra-observer reproducibility twice (short-term) at one day of temporal distance between the two replicas. These data were used for a preliminary assessment of inter-observer variability.

### Ethic statements

The study was conducted after the approval of the “Dipartimento di Scienze Cardiovascolari, Respiratorie, Nefrologiche, Anestesiologiche e Geriatriche, “Sapienza” Università di Roma and in accordance with the ethical guidelines of the Declaration of Helsinki. Written informed consent was obtained from each subject.

### Homologous times interpolation

**Figure 7:**
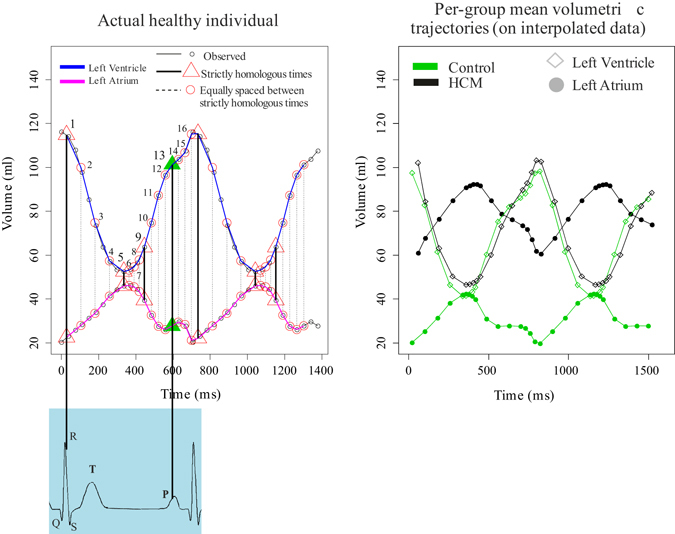
Interpolation procedure. The occurrence of homologous times (left) and mean volumetric trajectories of Control and HCM (right) are shown.

Each subject is acquired with a different number of frames depending on the frame rate, automatically set by the machine at each acquisition (mean frame rate: 41.6 ms), and on the beat rate (see Table 2). In order to study trajectory attributes, it is essential to evaluate shapes at physiologically homologous times. While in^8, 14, 16^ the set of homologous times refer to LV or LA only, in this study we needed to set them according to the concomitant function of both chambers. Figure 7 shows the concept of our procedure as applied to two heart beats.

We identified 4 strictly homologous times: two mechanical, LV end-systole and mitral valve opening, and two electrical, R wave peak and P wave peak. For each subject, we carefully inspected the 3DSTE video clip and we recorded the millisecond at which these events occur. Between consecutive strictly homologous landmarks we also estimated three equally spaced additional times. One single cycle comprises, thus, 16 homologous times. Then, we performed separate Generalized Procrustes Analyses (GPA) in the size and shape space (SSS) for the entire LH geometry taken as a whole shape on the shapes recorded for each individual and we interpolated them via cubic spline at the above mentioned homologous times. The use of SSS is justified here because at this step size is still maintained differently from successive analyses. The resulting dataset is constituted by 16 shapes, each composed by 2594 landmarks (1297 for LV and 1297 for LA), for any of the 66 subjects (46 Control and 20 HCM) under study.

### Left Heart trajectory

LH was treated as a whole shape and its motion was studied according to^8, 14, 16^. In order to deal only with pure deformations, we applied the PT geometrical tool. Basically, it estimates deformations within the cycle of each subject between observed shapes and a properly chosen local template (LT). Then all deformations are “transported” toward a common template (CT). After that a common GPA is performed. In this way, starting inter- individual shape differences are filtered out and the sole deformations can be studied. PT can be performed on the curved Riemannian manifold using sophisticated Riemannian geodesics estimation^38^ or on the Euclidean tangent plane to the CT by means of the so called “Linear Shift” (LS)^8, 14, 16, 39^ where geodesics are computed via simple subtraction and addition. It is important, however, properly managing rotations as explained in^38, 39^. In^8, 14, 16^, it was shown that LS approximation is fairly acceptable for the type of data used here and we thus adopted this strategy. It is important to note that LTs and CT were set as the local means of each individual and the grand mean of the entire sample, respectively. Further technical details about LS can be found in^8, 14, 16, 39^. After PT via LS, common ordination methods such as PCA can be performed. Motion trajectory is identified, for each subject, by the scores of the first three PCs. These will represent the “landmarks” of trajectory shape whose homology is ensured by the physiological temporal homology of times at which shapes were interpolated. Trajectory shape is studied by performing GPA + PCA on motions shapes; trajectory size is represented by trajectories centroid size (CS); trajectory angles are evaluated by computing the angle of vectors connecting PC scores values corresponding to end-diastole and those corresponding to end-systole. Both PC1/PC2 and PC1/PC3 angles are evaluated. These attributes were subjected to ANOVA (CS and angles) and MANOVA (shape) using Control/HCM as two-level factor variable.

It could be possible to use more than three PCs for building trajectories shapes but this would lead to the identification of hypershapes hardly representable. The first three PCs identify an elliptical trajectory shape and they explain a large amount of variance (see Results) thus making their use fairly acceptable.

As already highlighted in^8, 14, 16^ using local means as LTs and the grand mean as CT, allows appreciating both systole and diastole as deformed states. On the contrary, in fact, in classic 2D and 3D echocardiographic studies, end-diastole is a sort of “zero” from which deformations are computed. Our approach, instead, permits to identify possible impairments even if occurring during late diastolic phase.

### Hierarchical levels of covariation: strength and direction

Being LH organized into two highly interacting modules (LV and LA), the integration and covariation between them at different levels of organization can be studied. The first one is that occurring *within* each individual: the 16 LH shapes that constitute each individual cycle identify a specific covariation trajectory. All covariation trajectories can then be compared in terms of the strength and direction of covariation. One of the most used techniques to study the covariation between two blocks of variables (A and B, for example) is the Partial Least Squares Analysis (PLS)^40^. This approach treats blocks symmetrically without an a priori attribution of dependence-independence relationship. PLS returns two sets of *paired* singular vectors (SW) that maximize the covariance between blocks: one set for block A and one set for block B. These sets must be evaluated by plotting *pair by pair* the same SWs of both blocks. In the SW space the covariation trajectory can be evaluated in terms of strength and direction. Recently^29^ proposed a new strategy to compare the strength of PLS focusing on the first SW pair. They proposed a standardized test statistic (a z-score) for measuring the degree of morphological integration between sets of variables. The z-scores can be used to test for differences (via ANOVA) among groups. We then performed, on deformational data coming from LS procedure, separate PLS analyses for each individual using its proper 16 LV and LA shapes. We then used compare.pls() function from the R package “geomorph”^41^ in order extract z-scores (one value for each individual) to be used in ANOVA using Control/HCM as factor. This looks for differences in the strength of covariation, whereas nothing is known about its direction. In fact, in the SW space, two covariation trajectories could show the same strength in terms of effect size still having different orientations. The orientation of trajectory in SW space can be interpreted as the shape change rate of one module relatively to the shape change of the other. This aspect is very important as it is exactly related to the way the two modules (LV and LA) work in realizing LH functioning.

In order to investigate this issue, we performed separate Major Axis (MA) analyses on individual covariation trajectories on the space identified by the first pair of SWs. MA is necessary being it “symmetrical”, i.e. residuals are computed orthogonally to the line of best fit, and, coherently with the symmetry intrinsic to PLS, does not require the classic assumption of dependence-independence relationship. MA slopes are then contrasted in the usual ANOVA using Control/HCM as factor. A second-order level of covariation is that occurring between the shapes of trajectory themselves. In this case, we don’t analyze data *within* each individual, as one individual is represented by one single trajectory shape. Here covariation within any category (Control and HCM) is estimated using each individual trajectory shape as a single observation. LA and LV trajectory shapes were subjected to PLS analysis and the significance of difference in z-scores was evaluated via permutation analysis. Differences in direction were evaluated using MA regression as described above. This time, however, MA regression was performed on Control and HCM categories rather than on single individuals.

### Disintegration

Recently, in^42^ it was proposed a new, alternative way to look at the integration *intrinsic* to a special set of shapes. This approach does not compare two or more modules. Actually, it examines the regression coefficient for log partial warp variance against log of its proper bending energy in the standard thin-plate spline setup^41^. If the coefficient equals −1 the shape series can be considered as “self-similar” while if it is smaller the series is considered as “integrated”. When it results larger than −1 (e.g. close to 0) the series can be considered “disintegrated”, i.e. does not correspond to any biological meaningful explanation which is to say, incompatible with life. The more negative the coefficient the larger the integration. We thus performed such kind of analysis using globalIntegration() function in “geomorph” R package for each individual present in our dataset. Resulting slopes were subjected to ANOVA using Control/HCM as factor variable.

### Clinical classification performance

We used the indicators coming from LS analysis and trajectory analysis in a classification exercise in order to assess the clinical value of whole LH deformation as previously done in^8, 16^.

To achieve this we adopted the same strategy used in ref. 3 that consists in a randomized approach applied to Support Vector Machine (SVM) learning. In ref. 3 it was shown that SVM performs better than other common binary classification procedures (such as discriminant analysis and logistic regression, among others) in classifying myocardial infarction data very similar to those treated here. For this reason, we used SVM with radial basis Gaussian kernel function and hyperparameter C = 1 coupled with a randomized sub-data splitting. Briefly, we randomly choose 35 Controls and 17 HCM and we fit on them SVM for learning. Then we used the resulting coefficients for classifying the remaining 10 Control and 3 HCM. This is repeated 1000 times. Total accuracy, specificity, sensitivity and AUC from the ROC curves were calculated. Mean values are then reported. The above mentioned splitting (35/46 for Control and 17/20 for HCM) was forced by the small dataset we have here and by the necessity to use the majority of cases for learning. However, given the high number of randomizations we are confident that the performance indicators reported here well represent (if not underestimate) the actual classification power of LH deformation analysis. In addition, we showed results from the same analyses using the more traditional 3DSTE LV and LA strains in order to compare their performances with those of the new indicators built in this study. We also decided, in order to challenge our new approach, to include two variables that are not directly comparable to the ones we present here: LV mass and LA volume index. The former is affected by circular reasoning as it strongly depends on the thickness of interventricular septum that is used to diagnose the pathology; the latter is a purely size-connected parameter differently from our indicators that are calculated in the Shape Space (thus filtering out size).

## Electronic supplementary material

Supplementary Figure S1

Supplementary Figure S2
