# Supplementary figures and images for: Homeostatic Left Heart integration and disintegration links atrio-ventricular covariation’s dyshomeostasis in Hypertrophic Cardiomyopathy
Source: Sci Rep. 2017 Jul 24;7:6257. doi: 10.1038/s41598-017-06189-w (PMC5524707; doi:10.1038/s41598-017-06189-w)

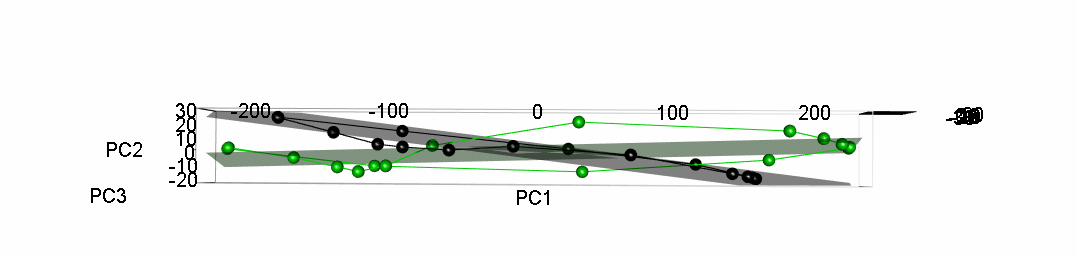

Supplement: Supplementary file 1 — Supplementary Figure S1 [file 41598_2017_6189_MOESM1_ESM.gif]

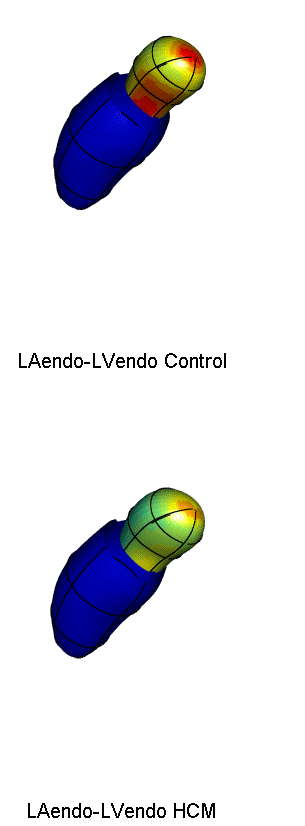

Supplement: Supplementary file 2 — Supplementary Figure S2 [file 41598_2017_6189_MOESM2_ESM.gif]
